# Targeting Bottlenecks in Malaria Transmission: Antibody‐Epitope Descriptions Guide the Design of Next‐Generation Biomedical Interventions

**DOI:** 10.1111/imr.70001

**Published:** 2025-02-05

**Authors:** Randy Yoo, Matthijs M. Jore, Jean‐Philippe Julien

**Affiliations:** ^1^ Program in Molecular Medicine The Hospital for Sick Children Research Institute Toronto Ontario Canada; ^2^ Department of Biochemistry University of Toronto Toronto Ontario Canada; ^3^ Department of Medical Microbiology Radboudumc Nijmegen The Netherlands; ^4^ Department of Immunology University of Toronto Toronto Ontario Canada

**Keywords:** antibody, malaria, vaccines

## Abstract

Malaria continues to pose a significant burden to global health. Thus, a strong need exists for the development of a diverse panel of intervention strategies and modalities to combat malaria and achieve elimination and eradication goals. Deploying interventions that target bottlenecks in the transmission life cycle of the causative agent of malaria, *Plasmodium* parasites, is an attractive strategy. The development of highly potent antibody‐based biologics, including vaccines, can be greatly facilitated by an in‐depth molecular understanding of antibody‐epitope interactions. Here, we provide an overview of structurally characterized antibodies targeting lead vaccine candidates expressed during the bottlenecks of the *Plasmodium* life cycle which include the pre‐erythrocytic and sexual stages. The repeat region of the circumsporozoite protein (CSP), domain 1 of Pfs230 and domains 1 and 3 of Pfs48/45 are critical *Plasmodium* regions targeted by the most potent antibodies at the two bottlenecks of transmission, with other promising targets emerging and requiring further characterization.

## 1 The Malaria‐Causing *Plasmodium* Parasites Have a Complex Life Cycle

The elimination and eradication of malaria has been a long‐sought goal in global health. Malaria affects approximately 250 million people annually with over 600,000 individuals succumbing to the disease—70% of them being children under the age of 5 years [1]. Although there are five species of *Plasmodium* known to infect humans, *Plasmodium falciparum* (Pf) is one of the most prevalant, accounting for the majority of cases and deaths [1]. Pf parasites are unicellular eukaryotic protozoans transmitted to humans through an *Anopheles* mosquito vector. While the use of various interventions such as insecticide‐treated nets and antimalarial drugs have aided in mitigating the spread and severity of the disease [2], progress in malaria elimination has stagnated in recent years [1]. Additionally, the emergence of insecticide‐ and drug‐resistant parasites calls for novel intervention strategies to be developed [3]. Of particular interest is the continued development of malaria vaccines. A major breakthrough in recent years is the World Health Organization (WHO) recommendation and prequalification for the use of two malaria vaccines, RTS,S/AS01 and R21/Matrix‐M [1]. While these vaccines have already saved lives and will continue to do so as their coverage expands, the immunity they induce wanes rapidly despite an already intensive administration schedule comprising three or four doses [4, 5]. Complementary vaccines such as those targeting other or multiple stages of the Pf life cycle, offer the potential to be utilized in conjunction with these vaccines, or next‐generation vaccines, to achieve disease control and global health goals [6, 7].

The *Plasmodium* life cycle can be broadly described as three stages that occur in the human host and mosquito vector: the pre‐erythrocytic, asexual blood, and sexual stages. In between these stages, the parasite undergoes significant changes in morphology, with a unique set of proteins expressed during each stage ‐ many of which being exclusively expressed during a single stage [8].

### 1.1 Pre‐Erythrocytic Stage

Malaria infection in humans begins with the pre‐erythrocytic stage when an *Anopheles* mosquito harboring *Plasmodium* sporozoites takes a blood meal. While the numbers can vary, it is estimated that approximately 10–10,000 sporozoites are inoculated into the skin during the meal [9, 10]. Sporozoites migrate through the dermis, seeking a blood vessel to enter the bloodstream [11, 12]. Once in circulation, they eventually reach the liver and interact with heparan sulfate proteoglycans expressed by a variety of cells that form the liver sinusoid [13]. A protein densely packed on the surface of sporozoites, circumsporozoite protein, mediates these interactions,C and parasite‐host cell binding signals the sporozoites to migrate across the sinusoidal barrier [14]. Sporozoites cross the barrier by either primarily traversing through Kupffer and endothelial cells, known as cell traversal [15], or paracellularly between them [16]. Movement is mediated by an actin/myosin motor linked to the extracellular protein thrombospondin‐related anonymous protein (TRAP) [17], which work in concert to achieve gliding motility critical for sporozoite infectivity [18, 19]. Cell traversal occurs through the invagination of the host cell membrane to form a transient intracellular vacuole that eventually fuses with a host lysosome [20]. Sporozoites escape degradation via the secretion of proteins found within parasite‐derived apical organelles, micronemes and rhotropies, which have been shown to contain proteins essential for traversal [21]. Cell traversal continues even when reaching the liver parenchyma. Sporozoites eventually stop traversing and engage in a productive invasion. Invasion has been shown to begin when a sporozoite protease cleaves CSP [22], allowing it to adopt an adhesive conformation [23], and binding to highly sulfated heparan sulfate proteoglycans [14]. This initiates the formation of a moving junction by rhotropy neck (RON) proteins and apical membrane antigen 1 (AMA1) [24] permitting entry into the host cell and the formation of the parasitophorous vacuole. The exact set of signals that drives the switch from traversal to productive invasion are not fully understood [21, 25]. The infected hepatocyte is remodeled to accommodate the first round of asexual reproduction resulting in the formation of a multinucleated schizont [26]. This process occurs over 6–7 days for Pf [27], but the time frame varies depending on species of *Plasmodium* [28]. The schizont contains thousands of merozoites which egress from the hepatocyte in the form of specialized vesicles, merosomes, into the sinusoid lumen [26, 29] and subsequently enter circulation.

### 1.2 Asexual Stage

The release of merozoites from merosomes signals the beginning of the asexual blood stage where they infect red blood cells (RBCs) in a span of minutes after hepatocyte egress [30]. The first primary merozoite attachment to a RBC is thought to occur through merozoite surface protein 1 (MSP‐1) binding to heparin‐like molecules [31], supported by interactions with RBC proteins glycophorin A [32] and band 3 [33]—the latter interaction having been shown to be essential. MSP‐1 is a proteolytically processed protein complex [34, 35] and the most abundant surface protein on merozoites. Alongside other GPI‐anchored as well as peripheral and integral membrane proteins, MSP‐1 forms a dense coat on the surface of the merozoite [36]. Subsequent binding events occur when specialized organelles, rhoptries, as well a distinct subset of micronemes are secreted. This results in the surface expression of various invasion‐related proteins such as those from the erythrocyte binding antigen (EBA) and reticulocyte binding‐like homologous (PfRH) protein families [37]. While redundant RBC binding pathways exist involving these proteins, the following step is conserved where parasite‐expressed PfRH5 binds to the RBC basigin protein receptor (CD147) [38, 39] anchoring the merozoite to the RBC. Finally, the parasite‐derived RON complex is inserted into the RBC membrane providing a receptor for AMA1 that is expressed on the parasite surface [40, 41]. This facilitates the formation of the moving junction that permits the entry of the merozoite into the RBC.

Following invasion, a parasitophorous vacuole membrane forms around the merozoite and dense granules are immediately released that deliver integral membrane protein Plasmodium translocon of exported proteins (PTEX) and ring‐infected erythrocyte surface antigen (RESA) to the parasitophorous vacuole membrane [42]. PTEX is a translocon [43, 44, 45] that mediates the export of proteins out of the parasitophorous vacuole membrane and is a key mediator of nutrient acquisition via RBC remodeling during the time the parasite resides in the RBC [46]. PTEX translocates RESA to the RBC cytosol where it then localizes to the intracellular side of the host RBC membrane via binding to spectrin [47] in a phosphorylation‐dependent manner [48]. Three distinct morphological stages follow spanning a total of 40–48 h [49]. The first stage, the ring stage, features a morphologically dynamic parasite alternating between amoeboid‐ and disc‐like shapes due to dense granule fusion with the parasitophorous vacuole membrane [42]. The trophozoite stage follows in which the parasite converts between ring‐like and irregular structures [50]. While the exact time frame is debated, approximately 40‐48 h after the invasion of the red blood cell, the parasite enters the schizont stage, increasing in size and taking on a ruffled appearance [50]. Multiple rounds of asexual reproduction give rise to 16–32 merozoites [28, 51] that release upon RBC rupture. Key proteins involved in these steps have been reviewed elsewhere [46].

### 1.3 Sexual Stage

A small subset of intraerythrocytic parasites, instead of producing asexual merozoites, will commit to gametocytogenesis marking the beginning of the sexual stage [52]. Sexual commitment likely occurs prior to schizogony in which all progeny in a schizont are primed to develop into either exclusively male or female gametocytes [53, 54, 55]. Pf gametocytogenesis takes over 9–12 days, the longest of all *Plasmodium* species [28]. Gametocyte maturation occurs in five stages with stages I–IV occurring in the bone marrow [56, 57]. During this time, key proteins integral to transmission biology are expressed. These proteins have been implicated in later parasite development in the mosquito midgut through studies utilizing various *Plasmodium* species. These include but are not limited to: Pfs230 [58], Pfs48/45 [59], Pfs47 (female‐specific) [60], and PfHAP2 (male‐specific) [61], which are all expressed on the parasite plasma membrane while residing inside the parasitophorous vacuole [8, 62, 63, 64]. While functions associated with these proteins are thought to be somewhat conserved between species, functional differences can exist [60, 65]. During this period, cytoskeleton modifications [66] and formation of an inner membrane complex [67] drive changes in RBC rigidity [68] and the formation of an elongated gametocyte shape. At stage V, a switch in deformability [68, 69, 70] coincides with the re‐entry of the RBC into circulation which provides an opportunity for RBCs harboring gametocytes to be taken up by a mosquito upon a blood meal.

When the RBCs enter the mosquito midgut, a drop in temperature [71] and the presence of xanthurenic acid [72, 73, 74] triggers gametogenesis. The high pH environment of the midgut has been proposed to act as a secondary signal [71]. Here, gametocytes emerge from their RBCs and female gametocytes mature into a single macrogamete, whereas male gametocytes will undergo three successive rounds of DNA replication to give rise to eight motile microgametes. The microgametes form exflagellation centers adhering to RBC surfaces [75, 76], where Pfs230 has been implicated [58]. Following this, a male microgamete adheres to a female macrogamete in which Pfs48/45 is predicted to play a role [59] and fusion of the two cells is mediated by eukaryotic fusogen, PfHAP2 [61, 77]. A zygote is formed that develops into a motile ookinete that travels from the midgut lumen across the epithelial layer, and lodges itself in the basal lamina to form an oocyst [75]. Several proteins play roles in protecting and supporting the development of the zygote, ookinete, and oocyst by enhancing survival in the mosquito midgut [78]. For instance, Pfs47 helps evade the mosquito immune system [79], and Pfs25 expressed during gametogenesis is implicated in protecting the parasite from mosquito‐derived proteases and aid in midgut traversal [80, 81]. In the oocyst, sporozoites are formed. After approximately 10–12 days, sporozoites emerge from the oocyst and migrate to the mosquito salivary glands where they are ready to infect a new human host.

## 2 Development of Next‐Generation Malaria Vaccines—Targeting Bottlenecks in the Malaria Life Cycle

Modeling suggests that the combination of a pre‐erythrocytic‐targeting vaccine and sexual stage‐targeting vaccine might be a cost‐effective route to lowering the burden of malaria through diminishing the infection–transmission cycle [6, 7, 82, 83]. These two stages represent bottlenecks in the Pf life cycle where essential surface‐expressed proteins with essential functions may be suitable antigens for vaccine development. Generally speaking, the antigens expressed during these stages exhibit low levels of polymorphisms [84, 85], hinting at conserved functions, suggesting that they are subject to limited selective immune pressure. Antibodies targeting the pre‐erythrocytic stage can stop infection outright and hence have been referred to as anti‐infective [86]. Monoclonal antibodies (mAbs) targeting this stage have increasingly been explored as biomedical intervention tools and emerging clinical data continue to solidify this strategy to reduce malaria [87, 88, 89, 90, 91, 92]. Antibodies that target sexual stage antigens and can reduce parasite transmission have been referred to as transmission‐blocking [93, 94]. Anti‐infection interventions can serve as a powerful tool toward reducing mortality and morbidity, and in combination with transmission‐blocking interventions can ultimately move us closer toward achieving elimination goals. Given these properties, lead vaccine antigens of these stages will be the focus of this review.

## 3 Targeting the Pre‐Erythrocytic Stage—Enhancing the Quality of the CSP Repeat‐Directed Response

**FIGURE 1:**
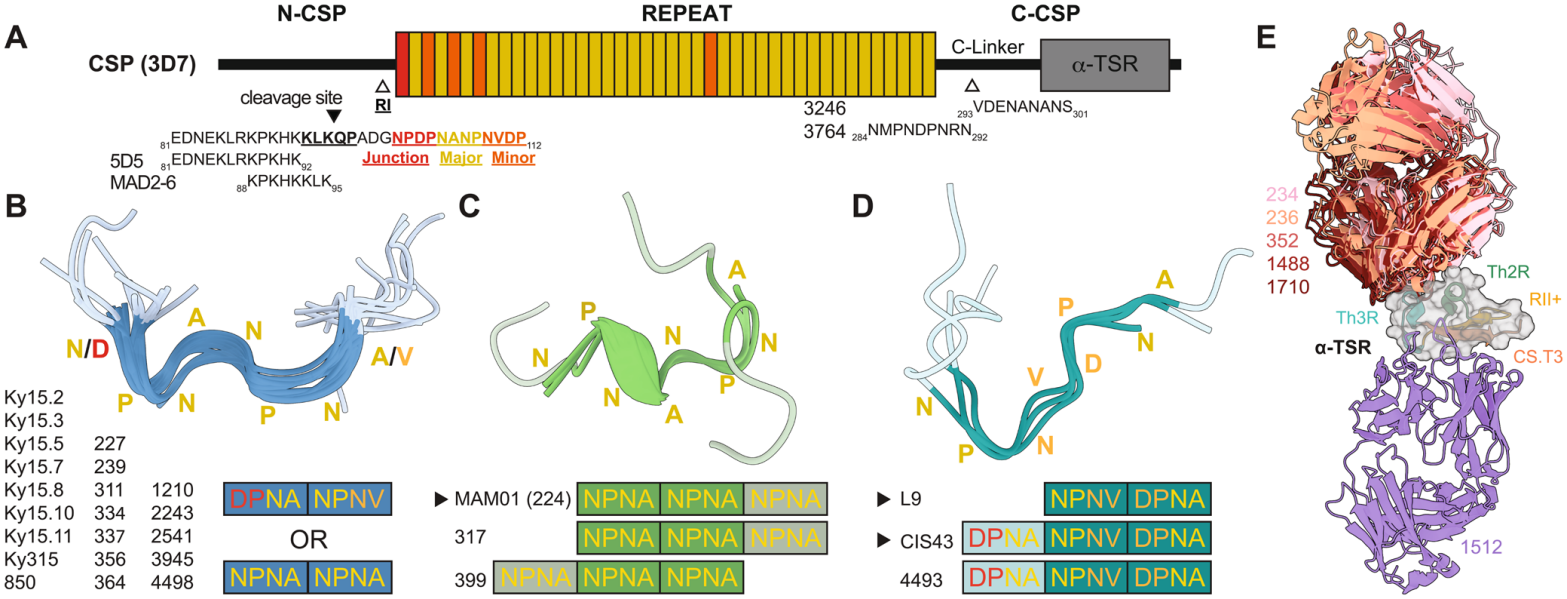
Overview of structurally characterized PfCSP‐directed mAbs. (A) The domain organization of PfCSP (3D7 strain) is shown as a schematic. The sequences of the downstream region of N‐CSP and a portion of the junctional region is shown. The sequence corresponding to region I (RI) is indicated in bold letters and underlined with the cleavage site in between the KQ residues indicated as a black arrow. The content of the different repeats found in PfCSP are indicated in bolded color and underlined. The junction motif NPDP is shown in red, the major repeat (NANP) in yellow, and the minor repeat (NVDP) in orange. The epitopes of structurally characterized N‐CSP‐directed antibodies (mAbs 5D5 and MAD2‐6) and C‐linker‐directed antibodies (mAbs 3246 and 3764) are shown below the schematic. Three CSP conformations of particular interest induced by antibody binding are (B) the 1210‐like (or inverted S) conformation in blue, (C) the 317‐like conformation in green, and (D) the U‐shaped conformation in teal. Shared secondary structural elements (turns or 3_10_ helix) are colored in darker colors with unique configurations of PfCSP colored in a lighter shade. Amino acid residues of the PfCSP repeats in the structure are indicated alongside the structures in accordance with the coloring scheme described in panel A. Antibodies denoted by a black arrow are those that have undergone clinical evaluation [87, 88, 89, 90, 91, 92]. (E) Structurally characterized mAbs that target the α‐TSR are overlaid with the α‐ctCSP binders colored in shades of red and mAb 1512 (β‐ctCSP binder) colored in purple. T cell epitopes, Th3R (cyan), Th2R (green), RIII+ (yellow), and CS.T3 (orange) are depicted on the structure.

CSP is a protein that is densely packed on the surface of pre‐erythrocytic sporozoites and plays a critical role in parasite invasion of hepatocytes [95]. The protein consists of three domains, the N‐terminal domain (N‐CSP), the central repeat region, and the C‐terminal domain (C‐CSP) (Figure 1A). On sporozoites, the central repeat region is highly immunogenic relative to the rest of the molecule [96, 97, 98, 99]. In Pf, the central repeat region consists of three types of tetrapeptide motifs, NPDP, NVDP, and NANP—the latter of which appearing the most frequently (Figure 1A). The number of tetrapeptide repeats present can vary across Pf strains, but the amino acid content of the repeats are highly conserved [85, 100]. This, in conjunction with the critical role of CSP in hepatocyte invasion, suggests that there is a low likelihood that this region will mutate drastically in response to immune pressure. TRAP is one of the few examples of a non‐CSP anti‐infection vaccine antigen that has been pursued as a vaccine candidate. TRAP, like CSP, has been demonstrated to be essential to sporozoite infectivity [19]. Various TRAP‐based vaccines have undergone clinical evaluation [101, 102]. Antibodies against TRAP have been shown to enhance the activity of antibodies against CSP, supporting the potential of exploring multiple antigens as vaccine candidates [103]. Although many surface‐presented proteins have been found to be expressed during the pre‐erythrocytic sporozoite stage, few have been as extensively characterized as CSP [86]. Given that the CSP‐directed humoral response has been studied much more thoroughly for the pre‐erythrocytic bottleneck, CSP will be the focus of this section.

The rationale for the selection of CSP as the basis of RTS,S was based on a series of immunization studies conducted beginning in the 1960s [104], whereby radiation‐attenuated sporozoite immunization protected rodents [105], primates, and human volunteers [106]. However, it was noted at the time that sporozoite‐based immunization may be impractical due to its poor stability and limited supply [107]. This motivated subunit vaccine development focused on CSP [99], as antibodies directed toward the central repeat domain neutralized the in vitro infectivity of sporozoites in a mouse–*P. berghei* infection model [108]. However, immunization with CSP alone did not generate sufficient clinical efficacy [109].

Therefore, drawing on the development of the *Energix‐B* vaccine against Hepatitis B, researchers utilized the hepatitis B surface antigen (HBsAg) as a carrier protein for the CSP central repeat region and C‐CSP. The C‐CSP region (Figure 1A) was included as it contains T‐ and B‐cell epitopes. Each letter in RTS,S links to a component in the vaccine: the “R” stands for repeat, as in the central repeat region of CSP; the T represents CD4 and CD8 T‐cell epitopes, Th2R and Th3R, respectively; finally, the “S”s stand for surface antigen of the Hepatitis B surface antigen. When expressed in yeast cells, one CSP‐fused HBsAg (RTS) tetramerizes with three HBsAg (S) monomers which were included to permit particle formation [110]. R21 differs from RTS,S in that it only consists of the CSP‐fused component, effectively presenting more CSP components. The deployment of these vaccines represents important milestones in malaria vaccine development. Since the initial design of these vaccines, a great amount of effort has been invested in delineating the contributions of antibody immunity to different regions of CSP.

### 3.1 Insights Into the CSP_Repeat_

To help better understand anti‐CSP antibody potency, Triller and Scally et al. [111] reported on the isolation of anti‐CSP antibodies elicited from donors from a malaria‐endemic region (Lambaréné, Gabon). In comparison with titers against a well‐defined asexual antigen, merozoite surface protein 3 (MSP3), anti‐CSP antibody titers were low in the sera of these individuals and only 45% of donors had circulating anti‐CSP antibodies. Twenty‐seven CSP specific mAbs were isolated from the two individuals who had the highest anti‐CSP titers. All isolated antibodies exhibited some degree of inhibition (20% or higher) in sporozoite hepatocyte traversal assays and some capacity to block hepatocyte infection in exoerythrocytic forms development assays. The antibodies that exhibited the highest inhibitory activity in the assays were selected for further characterization. These included IgG 663 and IgM 580. These antibodies made as IgG were able to protect mice in passive immunization studies using two different mouse models (C57BL/6 and FRG‐huHep [112]). Crystal structures of the germline variant of mAb 580 (580‐g) and mAb 663 in complex with an (NANP)_5_ peptide revealed that the antibodies induced an elongated and a turn conformation of the peptide, respectively. Structural analysis coupled with biophysical investigations revealed that somatic hypermutations were critical for high‐affinity binding. The rise in affinity of the matured antibodies compared to their germline were inferred to be a result of the stabilization of the antibody paratopes rather than introduction of new interactions as many of the residues that had undergone somatic hypermutation did not introduce interactions to the peptide antigen.

Concurrent to the structural insights acquired from this study, Oyen et al. [113] reported on the structures of two highly potent NANP‐targeting mAbs, 311 and 317, that were elicited in a controlled human malaria infection (CHMI) trial in which participants were vaccinated with RTS,S [114]. These mAbs were used in challenge studies using *P. berghei* expressing PfCSP (Pb‐PfCSP) [115] and C57BL/6 mice. Parasite liver load was assessed via qPCR for *P. berghei* specific 18S rRNA. mAbs 311 and 317 administered at a dose of 100 and 300 μg, respectively, inhibited parasite development effectively (97.2% and 99.7%, respectively) compared to a positive control mAb, 2A10, [97] administered at a dose of 300 μg (75%–82%). Crystal structures of these mAbs bound to a (NANP)_3_ peptide revealed that these mAbs induced a different NANP conformation distinct from mAbs 580‐g and 663 [111], showcasing the conformational flexibility of the NANP repeats (Figure 1B,C). Interestingly, the authors also performed negative stain electron microscopy using a recombinant CSP construct saturated with either mAbs 311 or 317 revealing that these Fabs bound to NANP repeats in close proximity to one another, inducing spiral‐like structures.

The repetitive nature of motifs within the CSP central region, and thereby the close proximity of repeating epitopes, offer the possibility that antibody–antibody contacts might be contributing to binding. Imkeller and Scally et al. [116] reported on the structures of two antibodies, mAbs 1210 (Figure 1B) and 1450, isolated from participants in a CHMI [98] that were vaccinated with live sporozoites under chemoprophylaxis (PfSPZ‐CVac) [117]. Structural studies revealed that these antibodies engage in antibody–antibody homotypic interactions in the antigen‐bound state. Interestingly, some of the residues involved in the homotypic interaction interface were evolved by somatic hypermutation. This suggests that B cells that had improved homotypic interaction were selected during affinity maturation. Through Ca^2+^ flux‐based B‐cell activation assays, it was demonstrated that B cells expressing 1210 B cell receptors (BCRs) activated more robustly than B cells expressing mutant 1210 BCRs that had perturbed homotypic interactions. This increase in activation was independent of affinity to a single NANP repeat epitope as isothermal titration calorimetry (ITC) experiments revealed a nonstatistically significant difference in binding affinity of mAb 1210 versus the 1210 mutant mAb to (NANP)_3_ which is too short to permit higher stoichiometric binding. Interestingly, perturbing the homotypic interaction interface did not affect the protective efficacy of the antibodies when passively transferred to C57BL/6 female mice and challenged with Pb‐PfCSP. This finding suggested that homotypic interactions might be more relevant in the context of B cell responses to repeat antigens than in the context of antibody function, that is, parasite inhibition. This contrasts with what was observed in a follow‐up study on mAbs 311 and 317 by Martin et al. [118] who solved and analyzed cryogenic electron microscopy structures of mAb 311, mAb 317 [119], and other mAbs [119] (Figure 1B–D) bound to recombinant CSP containing 19 NANP repeats [120]. These mAbs, all isolated from individuals in a RTS,S clinical trial [114], engage in homotypic interactions when bound to CSP. Perturbing the homotypic interaction interface of these mAbs resulted in lower protective efficacy in liver burden assays in a statistically significant manner. The same reduction was observed when mutating out the homotypic interface of L9 [121] (Figure 1D), which is selective toward the minor repeats of CSP [121, 122, 123] (Figure 1A). More work is therefore needed to fully understand the role of homotypic interactions in the CSP‐directed immune response and how these might shape B cell maturation and protective efficacy.

### 3.2 CSP_REPEAT_ —Junction‐Targeting mAbs

In a continued effort to discover potent mAbs, Kisalu and Idris et al. [124] investigated the sera of volunteers in a CHMI trial who were vaccinated with an attenuated whole‐sporozoite vaccine [125]. One of the mAbs isolated, CIS43, consistently exhibited the most potent properties across various assays. In one of the assays performed, CSP‐directed mAbs were passively transferred to C57BL/6 mice, which were subsequently challenged with Pb‐PfCSP [115] through intravenous injection or through infectious mosquito bite. In experiments where parasites were injected intravenously, CIS43 consistently outperformed NANP‐directed positive control mAb, 2A10 [97], at reducing parasite liver burden in mice (assessed via qPCR for *P. berghei*‐specific 18S rRNA) in a statistically significant manner. The same trend was observed in the mice challenged via mosquito infection in that all 14 mice that received CIS43 were free of parasites 12 days post infection. In contrast, only two of seven mice receiving mAb 2A10 [97] were parasite free. Series of enzyme‐linked immunosorbent assay (ELISA), isothermal titration calorimetry, and X‐ray crystallography experiments revealed that CIS43 preferentially bound to the newly defined junctional epitope over the NANP repeats (Figure 1D). The junctional epitope of CIS43 consists of residues NPDPNANPNVDPNAN and is derived from the region between N‐CSP and the central NANP repeats (Figure 1A).

Concurrent to the discovery of CIS43 [126], Tan et al. [125] reported on the isolation of multiple mAbs from individuals who were vaccinated with an attenuated whole‐sporozoite vaccine. Many of the mAbs were not only junction‐preferring binders but also cross‐reactive to the NANP repeats [127]. A crystal structure of one of these mAbs, MGG4 [127], in complex with a junction‐derived peptide revealed the antibody targeted an epitope centered around the DPN motif in the N‐junctional region, similar to CIS43 [127]. These discoveries highlighted how junction‐directed mAbs could greatly contribute to a potent immune response, as suggested by previous reports [128, 129]. This primed others, such as Wang et al. [123], to continue searching for additional junction‐targeting potent mAbs such as L9. Elicited by whole‐sporozoite immunization [125], L9 was determined to be a potent minor repeat preferring binder, targeting the NANPNVDP minor repeats (Figure 1D) while maintaining a small degree of cross‐reactivity to NANP repeats.

To gain additional insights into structural determinants responsible for cross‐reactive binding, Murugan, Scally, and Costa et al. [130] isolated and characterized 155 CSP‐directed mAbs elicited in malaria‐naïve individuals that were immunized repeatedly with live sporozoites under chemoprophylaxis (PfSPZ‐CVac) [117]. Using ELISA experiments, the reactivity of these mAbs were tested against five peptides/constructs consisting of different regions of CSP. These included three peptides consisting of overlapping regions in the N‐terminal junction, (KQPADGNPDPNANPN, NPDPNANPNVDPNANP, and NVDPNANPNVDPNANPNVDP), one peptide representing the NANP repeats [NP(NANP)_5_], and a peptide of the linker between the central repeat and C‐CSP, which contains a NANA motif. Fifty‐nine percent of the isolated antibodies displayed some degree of cross‐reactivity, binding to at least two of these targets. Five mAbs with distinct cross‐reactivity profiles, but primarily selective toward the NANP peptide, were selected for structural studies. Four of these mAbs (2243, 2541, 3945, and 4498) were co‐crystalized with an NANP repeat‐derived peptide and one of these mAbs (3246) was co‐crystalized with the NANA peptide derived from the C‐terminal linker of CSP. Like the previously reported mAb 1210 [116], these mAbs bound to their respective peptides in an inverted S conformation (Figure 1B). The electrostatic properties of the antibodies paratopes provided a molecular basis for cross‐reactivity. For instance, mAbs 1210 [116] and 2243 [130] disfavored binding to the negative charge‐containing NVDP junction peptides in isothermal titration calorimetry experiments due to the electronegative patches in their paratopes. In contrast, mAbs 2541, 3945, and 4498 were able to accommodate binding to the minor repeat peptides, likely due to the less electronegative and polar nature of their paratopes.

Murugan, Scally, and Costa et al. [130] also structurally characterized mAb 4493 which differed from the other mAbs as it was cross‐reactive but slightly preferring the NPDPNANPNVDPNANP peptide. Crystal structures of mAb 4493 in complex with various peptides derived from the junction region and NANP revealed that the mAb bound to all peptides nearly identically [root mean squared deviation (r.m.s.d.) < 0.2 Å]. The slight preference toward the NPDPNANPNVDPNANP peptide was due to a more extensive H‐bonding network not recapitulated when bound to other peptides. Interestingly, the conformation the peptides adopted was reminiscent of what was observed in the CIS43 [126] crystal structures (Figure 1D), although the angle of approach differed. C57BL/6 mice passively immunized with mAb 4493 [130], CIS43 [126], or NANP‐selective mAb 317 [113] and challenged with Pb‐PfCSP [115] via mosquito bite were protected from infection to varying degrees when administered at 150 or 300 μg. Although differences were observed, they were not statistically significant. This suggests that both the junctional and major repeat are epitopes targeted by potent antibodies.

Thai and Murugan et al. [131] expanded on these molecular insights when structurally characterizing 12 CSP‐specific mAbs elicited in Kymice [132]. Kymice, which carry human immunoglobulin (Ig) loci, were immunized with nanocage‐based immunogens containing junctional, minor, and major PfCSP repeat motifs [133]. Thai and Murugan et al. [131] focused on IGHV3‐33 mAbs as they are highly selected for in the anti‐PfCSP humoral response [98, 113, 116, 126, 130, 134]. Twelve mAbs were isolated and crystalized in complex with the following peptides: KQPADGNPDPNANP, NPDPNANPNVPDNANP, NVDPNANPNVDP, and NANPNVDPNANP, (NANP)_3_, and (NANP)_5_ for a total of 22 crystal structures. Strikingly the majority of mAbs (11/12), irrespective of which peptide they were bound to, induced an Asx turn (Asn pseudo 3_10_ turn) in the C‐core. An HCDR3‐encoded tyrosine residue was commonly observed to stack against this turn. Excluding Ky224 and Ky230, the mAbs adopted one of two configurations in the N‐core: the 1210 [116]‐like (Figure 1B) or MGG4 [127]‐like mode of binding. The former configuration 1210‐like (or inverted S), is also observed in the context of many other mAbs [113, 116, 119, 120, 130, 135]. This conformation was associated with higher cross‐reactivity and potency, attributed to a more compact binding interface which conferred higher affinity. Ky224 and Ky230 were distinct in that neither adopted these conformations. Interestingly, the conformation of CSP adopted in the Ky230‐bound state is similar to what is seen in the co‐crystal structure of non‐potent, CIS42, in complex with an NANP peptide ‐ both of these antibodies being poorly potent. This suggests that this conformation may not be conducive to potent sporozoite neutralization. Unlike Ky230, however, Ky224 performed on par with the 1210‐like binders at conferring liver burden reduction or protection from parasitemia [136] despite only being selective toward one peptide, NPDPNANPNVPDNANP. Overall, the commonly observed conformations of CSP induced by potent antibodies are similar in that they feature sequential secondary structural elements (β‐turn variants or a 3_10_ helix) (Figure 1B–D) which has been discussed previously by Pholcharee et al. [119]. Together, these data highlight how certain types of antibody binding modes are enriched in the response and how a diversity of structural solutions exist to achieve mAb potency against CSP repeat motifs (Figure 1B–D).

### 3.3 N‐CSP

The N‐CSP region has been implicated in various roles for sporozoite development and infectivity. Namely, a region encompassing Region I (RI) (LRKPKHKKLKQPADG, Region I: KLKQP) [137] (Figure 1A) mediates localization of sporozoites to the mosquito salivary gland [138]. Myung et al. [137] found the upstream lysine residues were integral to this process. In addition to this, RI has been implicated to be cleaved by a parasite‐derived cysteine protease [22] during productive invasion triggered through heparan sulfate proteoglycans binding [14]. Recently, it has been uncovered that cleavage occurs between the lysine and glutamine residues of RI, resulting in a post‐translational modification of the N‐terminal glutamine to cyclize, yielding a pyroglutatmic acid moiety that can be recognized by inhibitory antibodies [139]. Finally, the *Plasmodium* export element (PEXEL) cleavage sites found upstream of RI have been suggested to play intracellular roles in liver hepatocyte development after productive invasion of hepatocytes [140]. Altogether these indicate that the N‐CSP region may play critical roles in the *Plasmodium* life cycle and potentially, perturbing these processes could contribute toward inhibition of sporozoite development.

To date, very few mAbs against N‐CSP have been isolated or characterized. Thai, Costa, and Weyrich et al. [141] reported on an N‐CSP‐directed mAb, 5D5, a murine mAb elicited via immunization with full‐length recombinant CSP [142, 143]. Thai, Costa, and Weyrich et al. [141] solved a crystal structure of mAb 5D5 in complex with an N‐CSP‐derived peptide in which residues 81–91 could be resolved (^81^EDNEKLRKPKH^91^) (Figure 1A). Interestingly, the mAb leverages a glycan present in its HCDR3 to facilitate binding. Despite the epitope that mAb 5D5 targets being present in mosquito‐derived sporozoites, the mAb was found not to effectively bind sporozoites derived from the mosquito midgut or salivary glands. In line with this finding, delivery of single chain Fab (scFab) variants to *A. coluzzii* female mosquitos revealed that repeat‐targeting scFab1210 could significantly reduce the number of mature sporozoites in the salivary glands while the scFab5D5 did not. Finally, passive immunization studies of mice injected with repeat‐targeting mAb, 1210, or mAb 5D5 challenged with Pb‐PfCSP [115, 136] revealed that mAb 5D5 was not as effective as mAb 1210 at reducing parasite liver burden in a statistically significant manner.

Tan, Cho, and Pholcharee et al. [144] reported on N‐CSP‐directed mAb, MAD2‐6. This IgA mAb was elicited from natural parasite exposure in a Malian individual enrolled in a longitudinal observational cohort study [145]. C57BL/6 mice passively immunized with MAD2‐6 IgA was more effective at reducing parasite liver burden when challenged with Pb‐PfCSP [115, 136] compared to an untreated control in a statistically significant manner. However, it was not as effective as potent N‐junction binder, CIS43 [126]. Interestingly, MAD2‐6 was better at binding mosquito midgut‐derived sporozoites compared to ones from the salivary gland. Crystal structures of MAD2‐6 in complex with _88_KPKHKKLKQ_96_‐containing peptides revealed that the MAD2‐6 epitope directly overlaps with RI and partially overlaps with the downstream portion of the epitope targeted by 5D5 [141] (Figure 1A). Interestingly, MAD2‐6 participates in homotypic interactions in which each Fab paratope is centered on two lysine residues separated by two residues (KxxK, K90 + K93 and K92 + K95). The authors note that this is striking given that the motif is very similar to heparin/heparan sulfate consensus binding motifs and did confirm that MAD2‐6 and heparin compete for binding to the peptide containing the MAD2‐6 epitope.

Currently, to the best of our knowledge, no mAb targeted to elements upstream of the RI cleavage site rival the potency of the best junction/repeat‐directed mAbs. Future work may lead to a better understanding of how N‐CSP‐directed mAbs contribute to the CSP‐directed humoral response.

### 3.4 C‐CSP

The C‐terminal domain of CSP (C‐CSP) consists of a C‐linker region and an α‐thrombospondin type‐1 repeat (α‐TSR) domain, acting as a tether point to the sporozoite membrane through a glycosylphosphatidylinositol (GPI)‐anchor [146]. Antibodies against both the C‐linker region and the α‐TSR domain have been reported on more extensively in comparison with N‐CSP.

Interestingly, CSP‐directed mAbs have been isolated that are cross‐reactive to C‐CSP and the repeat region of CSP. Murugan, Scally, and Costa et al. [130] solved a structure of mAb 3246 in complex with a NANA peptide (PNRNVDENANANSA) representing the C‐linker region (Figure 1A). This mAb recognizes the NANA peptide and an NP(NANP)_5_ peptide with comparable affinity levels in surface plasmon resonance (SPR) experiments. In this study, mAb 3945 [130], which was crystalized with an (NANP)_5_ peptide but was cross‐reactive to the NANA peptide, also harbored a small serine side chain residue in its KCDR3 that is occupied by a tyrosine in the non‐C‐linker‐reactive mAbs, suggesting this residue position may drive NANA cross‐reactivity in the 1210‐like binders. Oludada et al. [147], isolated and characterized C‐CSP‐directed mAbs from malaria‐naïve European volunteers who were immunized with radiation‐attenuated sporozoites [147, 148]. mAb 1961, isolated from one of these individuals, displayed cross‐reactivity to C‐CSP, NANP_10_, and an N‐junction peptide (GKQPADGNPDPNANPNVDPNANP) in ELISAs. In follow‐up experiments utilizing overlapping peptide regions of C‐CSP, the specific region on C‐CSP bound by mAb 1961 was determined. mAb 1961 only displayed binding to the same NANA peptide (PNRNVDENANANSA) that mAbs 3246 [130] and 3945 [130] were cross‐reactive to, suggesting a similar mode of recognition, although mAb 1961 was derived from an IGHV3‐21 heavy chain in contrast to the IGHV3‐33 of mAbs 3246 [130] and 3945 [130]. Despite these interesting properties, mAb 1961 did not effectively protect C57BL/6J mice challenged with bites from Pb‐PfCSP‐infected mosquitoes from developing parasitemia compared to NANP‐directed mAb 317 [119].

In the Oludada et al. [147] study, another mAb was found to target the C‐linker region: mAb 3764 (Figure 1A). Through ELISAs, mAb 3764 was determined to bind to a region (QGHNMPNDPNRNVD) upstream of and that partially overlaps the binding site of mAb 1961. A crystal structure of mAb 3764 in complex with the peptide corresponding to this region revealed that the epitope was centered on a DPN motif, also found in the junctional region of CSP. However, mAb 3764 was not cross‐reactive toward an N‐junction peptide (GKQPADGNPDPNANPNVDPNANP) in ELISA nor in SPR experiments. Molecular modeling predicted that the lack of cross‐reactivity was owed to a loss of significant interactions and introduction of steric hindrance upstream of this DPN region when replacing C‐CSP linker residues with N‐junction residues in the corresponding positions. Despite these interesting molecular insights, mAb 3764 only displayed weak binding to sporozoites at high concentrations (100 μg/mL) and failed to inhibit Pf sporozoite traversal [149] at the same concentration.

In comparison with the linker region, many more antibodies targeting the α‐TSR region of C‐CSP have been structurally characterized. Scally and Murugan et al. [150] reported on mAb 1710, which was isolated from a European volunteer immunized with cryopreserved live Pf sporozoites under chloroquine prophylaxis (PfSPZ‐CVac) [117]. The crystal structure of mAb 1710 in complex with a recombinant α‐TSR construct revealed that the antibody bound to a conformational epitope consisting of two T cell epitopes found in this domain, Th2R and Th3R (Figure 1E). Two other T cell epitope exist in this domain, RII+ and CS.T3, which are localized to the other side of the α‐TSR domain [151] (Figure 1E). mAb 1710 bound poorly to Pb‐PfCSP sporozoites in live immunofluorescence and flow cytometry experiments at high concentrations (100 μg/mL) compared to NANP‐directed and positive control mAb, 2A10 [97]. The poor recognition of sporozoites translated to poor Pb‐PfCSP sporozoite traversal inhibition permitting the development into exoerythrocytic forms. Additionally, mAb 1710 provided poor protection in vivo. Only a small number of C57BL/6 mice passively immunized with 400 μg of 1710 IgG were free of blood stage parasites after being challenged with Pb‐PfCSP sporozoites. This is in contrast to mice injected with 400 μg of positive control, NANP‐directed mAb, 663 [111], which protected all mice from developing blood stage parasites.

Beutler, Pholcharee, and Oyen et al. [114] found that 1710‐like mAbs were elicited in the participants enrolled in the phase 2a RTS,S trial when they structurally characterized C‐CSP mAbs [152]. Four mAbs (mAbs 234, 236, 352, and 1488) were crystalized in complex with an α‐TSR construct (ctCSP) revealing that they all bound the same epitope as mAb 1710 with very similar angles of approach (Figure 1E). Beutler, Pholcharee, and Oyen et al. [152] also structurally characterized a mAb that bound to the opposite side of α‐TSR, mAb 1512, which forms contacts with T cell epitopes RII+ and CS.T3 (Figure 1E). The authors aptly named this face the beta epitope (β‐ctCSP) as it primarily consists of β‐strands. In contrast, the 1710‐like binding mAbs (mAbs 234, 236, 352, and 1488) target a surface comprising of primarily helices that comprise the Th2R and Th3R T cell epitopes and was thus named the alpha epitope (α‐ctCSP) (Figure 1E). Unlike the alpha epitope, the beta epitope is highly conserved across various strains of Pf and mAb 1512 alongside other alpha epitope binders identified in the study exhibited nanomolar‐level binding in SPR experiments when binding to 15 different haplotype variants of ctCSP. In contrast, the 1710‐like binders (mAbs 234, 236, 352, and 1488) exhibited poor breadth, only binding to the RTS,S matched strain (3D7) ctCSP and to only one (mAbs 236, 352, and 1488) or three (mAb 234) other haplotypes out of the 15 tested. In a challenge model utilizing C57Bl/6 mice and Pb‐PfCSP [136, 153], alpha epitope‐targeting mAb, 236, and beta epitope‐targeting mAb, 512, both inhibited liver parasite burden more effectively than the naïve control group in a statistically significant manner. However, Oludada et al. [147] showed how both alpha and beta epitope‐directed mAbs elicited by immunization with radiation‐attenuated sporozoites [148] could not effectively bind to sporozoites in flow cytometry experiments at high concentrations of 100 μg/mL. Additionally, they exhibited poor Pb‐PfCSP sporozoite traversal inhibition in comparison with positive control mAb, 2A10 [97]. Similar findings have been observed in an independent study by Wang et al. [154] in that C‐CSP‐directed mAbs were poor binders and neutralizers.

Altogether, these data exemplify that while C‐CSP‐directed mAbs with in vivo inhibitory capabilities could be identified (mAbs 236 and 1512) [152], the majority are poor neutralizers in comparison to repeat‐directed mAbs [147, 150, 154]. C‐CSP, while being less immunogenic than the repeat region when presented endogenously on the surface of the parasite [96, 97, 98, 99], is highly immunodominant when immunized into C57BL/6J mice as recombinant soluble full length (FL) PfCSP [133]. Sera from these mice showed binding to Pf sporozoites, which was reduced when the sera was depleted of the NANP‐directed antibodies [133]. In line with reduced sporozoite binding, NANP‐directed antibody‐depleted sera had a reduced capacity to inhibit Pf sporozoite traversal of hepatocytes in vitro [133]. While it has been reported that antibodies directed against C‐CSP mediate phagocytosis, complement fixation, and opsonization in vitro [155, 156, 157, 158, 159, 160], the polymorphic nature of C‐CSP may significantly reduce the breadth of a vaccine‐induced response. Additionally, the C‐CSP region might not be readily accessible on sporozoites [147, 150, 154]. While the presence of repeat‐directed mAbs may ameliorate this apparent inaccessibility, it does not appear to translate into improved inhibition [154]. Thus, despite reports of C‐CSP titers correlating with protection [157, 161, 162, 163], there is a current lack of evidence that C‐CSP mAbs are on par in terms of potency and epitope conservation compared to junction/repeat mAbs, which therefore puts into question whether the C‐CSP should be prioritized in biomedical interventions seeking potent inhibitory responses.

### 3.5 Integrating Molecular Insights Into Immunogen Design

Ludwig, Scally, and Costa et al. [133] sought to leverage these mAb structure–function relationships to design immunogens that preferentially elicit antibodies that target the most potent epitopes on PfCSP. N‐ and C‐CSP domains were excluded and PfCSP residues 95–148 (Uniprot: P19597) were fused to the N‐terminus of *H. pylori* apoferritin. This PfCSP region contains the native junction region starting at KQPA followed by the minor repeats and only five consecutive NANP repeats. The number of NANP repeats were limited to avoid the potential activation of low‐affinity B cells by strong BCR cross‐linking that is hypothesized to dampen the potency of the humoral response [164]. To re‐instill the ability to efficiently recruit T cell help after excluding the PfCSP‐derived T cell epitopes in C‐CSP, a universal pan DR T helper cell epitope, PADRE, was fused to the C‐terminus of *H. pylori* apoferritin. The resulting immunogen self‐assembles into spherical nanocages with 24 subunits which presents PfCSP on the surface while the PADRE T cell epitope is encapsulated internally, shielding it from the humoral response.

Pooled sera from Kymice immunized with this immunogen, 126, showed potent hepatocyte traversal inhibition of Pf sporozoites in vitro in comparison with FL‐PfCSP in a statistically significant manner [133]. A modified immunogen, 145, was designed to include two non‐native N‐linked glycosylation sites on the *H. pylori* apoferritin protomer (thereby 48 engineered N‐linked glycosylation sites for fully assembled apoferritin nanocages) in an effort to lower scaffold‐directed antibodies [165, 166]. High mannose glycoforms were achieved via expression in HEK 293S cells [GnT I^−/−^] (immunogen 145S). Mice immunized with immunogen 145S yielded sera with lowered anti‐scaffold responses compared to immunogen 126 while also enhancing the PfCSP‐directed response. This translated into more potent Pf sporozoite traversal inhibition in vitro in a statistically significant manner. Mice immunized in a prime‐boost regimen with immunogen 145S, challenged with bites from Pb‐PfCSP‐infected mosquitoes were free from blood stage parasitemia 23 days after the booster immunization. This protection was durable in that even at ~5 months post‐immunization (day 142), 3/5 or 4/5 mice were protected over a 20‐fold dose range (10 and 0.5 μg of immunogen 145S, respectively). Ludwig, Scally, and Costa et al. [133] hypothesized that the dampening of the anti‐scaffold response and boosting of the anti‐PfCSP response through N‐linked glycan engineering was likely due to the utilization of the mannose‐binding lectin (MBL) and complement pathway to improve the antigen‐specific humoral response through trafficking antigen to germinal centers, as had previously been established for other antigens [167, 168]. Future studies will help enhance our understanding of how glycosylation, in addition to precise mAb structure–function relationships, nanocage engineering and T cell epitope optimization, can contribute to shaping the immune response to PfCSP and other malarial antigens.

## 4 Targeting the Sexual Stage—Transmission‐Blocking Vaccines

Transmission‐blocking vaccines (TBVs) act by eliciting antibodies against sexual stage antigens that perturb the fertilization process that occurs in the mosquito vector, subsequently blocking transmission. These antibodies, which circulate in the bloodstream, are taken up during a mosquito blood meal and bind to parasites when they emerge from the red blood cells in the mosquito midgut. The concept was demonstrated by Gwadz [169] and Carter and Chen [170] in which attenuated parasites or purified gametes, respectively, were used to immunize chickens. Mosquitos that had taken up sexual stage parasites and antibodies from these chickens were largely protected from parasite oocyst development. While *Plasmodium* gametocyte antigens remain hidden from the immune system when residing inside red blood cells, antibodies against these antigens have been shown to be acquired in natural infection [171].

While many TBV candidates have been proposed [172], considerable efforts have been deployed on Pfs25. From a structural perspective, Scally et al. [173] characterized anti‐Pfs25 mAbs elicited in Kymice [132] via recombinant plant‐produced Pfs25 virus‐like particles (VLPs) [174] immunization. Following this, Mcleod et al. [175] and Shukla et al. [176] each structurally characterized human mAbs isolated from clinical trial participants that were immunized with Pfs25‐VLP or Pfs25 conjugated to Exoprotein A (Pfs25‐EPA), respectively. While these findings provided important molecular insights into mAb potency, higher order preclinical species and clinical‐stage data on Pfs25‐based immunogens revealed that they did not induce sufficiently high transmission‐blocking sera or did not induce a durable response [174, 177, 178, 179]. Healy et al. [179] compared immunogens based on Pfs25 to a domain 1 fragment of Pfs230 (Pfs230D1) consisting of residues 542–736 (Pfs230D1M) (Uniprot: P68874). Both antigens conjugated to EPA elicited similar levels of transmission‐reducing sera in BALB/c and CD‐1 outbred mice. However, in immunization studies in rhesus macaques and vaccination in humans, transmission‐reducing activity (TRA) correlated well with the anti‐Pfs230 but not the anti‐Pfs25 response [179]. Mirroring what had been previously described [180, 181, 182, 183], activity of the sera was enhanced via complement as heat‐inactivated sera resulted in a decrease in TRA.

**FIGURE 2:**
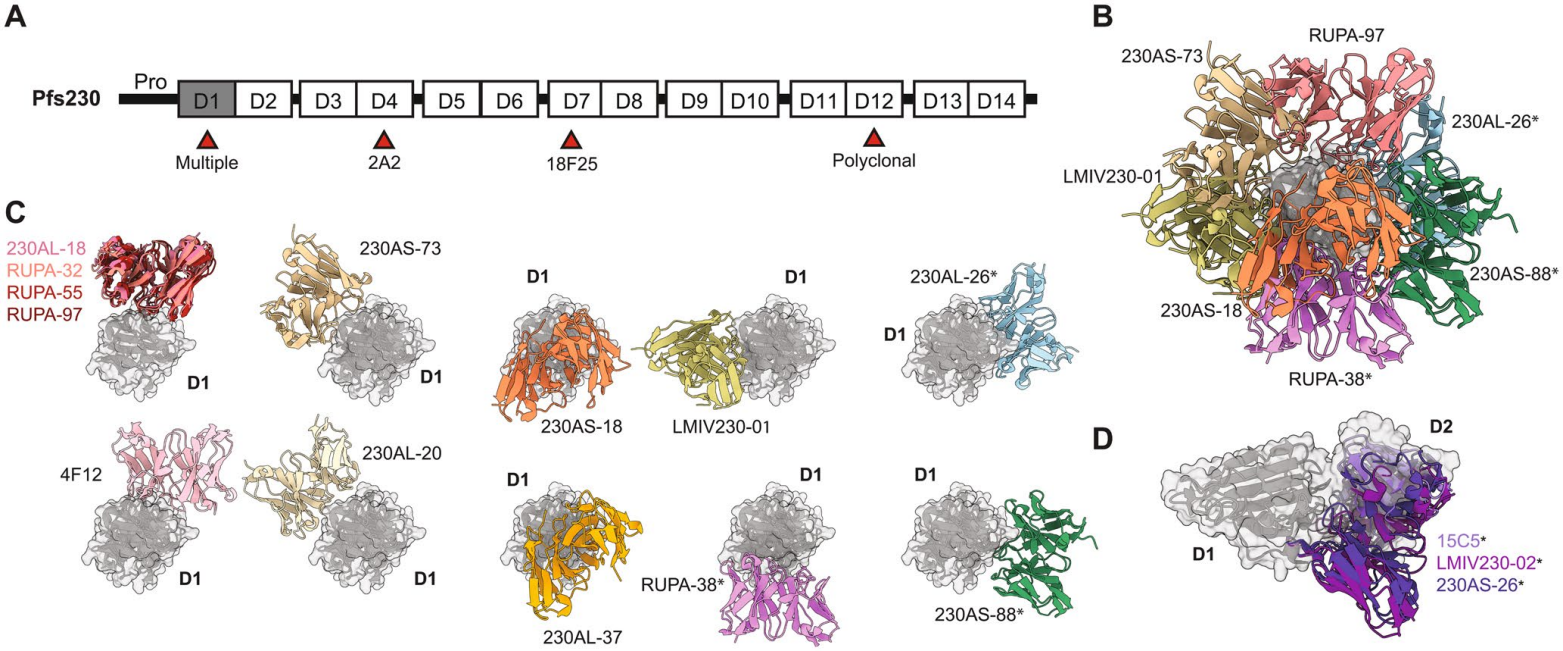
Overview of structurally characterized Pfs230D1‐directed mAbs. (A) The domain architecture of Pfs230 is shown with boxes indicating each 6‐Cysteine domain (not to scale). The Pro region that is proteolytically cleaved off [185] is present upstream of Pfs230D1 (gray). Domains that have been reported to elicit potent antibodies are indicated with a red arrow. (B) General overview of the various epitopes targeted by representative Pfs230D1‐directed mAbs with Pfs230D1 in gray. (C, D) All currently available structures of Pfs230D1‐directed mAbs in complex with Pfs230D1 (gray). (C) All non‐bin III binders are shown. The potent bin I binders are overlaid and colored in different shades of red. Bin II antibodies consist of mAbs LMIV230‐01 (olive) and RUPA‐38 (magenta). (D) All bin III binders are colored in different shades of purple. Asterisks next to the antibody indicates that they are poorly inhibitory (IC_80_ > 100 μg/mL).

Pfs230 is a protein of approximately 230 kDa and is anchored to the gametocyte surface through a non‐covalent interaction to Pfs48/45 that is GPI‐anchored to the parasite membrane [184]. The protein consists of 14 (Figure 2A) 6‐Cysteine domains [186] which are Immunoglobulin (Ig)‐like folds containing up to six cysteines. Structural insight into Pfs230 is currently limited to just the first two domains in which a prototypical A‐type on B‐type fold [186, 187, 188] is observed. The majority of functional antibodies that have been described target Pfs230D1 [189, 190, 191]. However, other non‐Pfs230D1‐targeting antibodies such as the murine‐derived 2A2 [181, 192] and 18F25 [193, 194], also display high TRA with an IC_80_ of 1.9 μg/mL [195] and TRA = 93% at 30 μg/mL [196], respectively (Figure 2A). Additionally, murine antibodies raised against domain 12 of Pfs230 displayed high levels of potency [197] (Figure 2A). However, structural characterization of Pfs230‐directed mAbs has been limited to those that target Pfs230D1.

### 4.1 Antibodies Against Pfs230

The potency of transmission‐blocking mAbs are evaluated in standard membrane‐feeding assay (SMFA) experiments. SMFAs are best interpreted when TRA of 80% or higher is achieved [198]. Thus, IC_80_ values (concentration of mAb required to achieve 80% TRA) are commonly reported. For this review, IC_80_ values or the lowest concentration of mAb tested that achieves TRA higher than 80% are referenced.

LMIV230‐01 is a mAb that was isolated from a participant in a clinical trial where the Pfs230D1M‐EPA vaccine was evaluated [199]. In this study, LMIV230‐01 exhibited ~80% TRA at 60 μg/mL in SMFAs which was reduced when heat‐inactivated sera lacking complement activity was used. A crystal structure of LMIV230‐01 in complex with Pfs230D1 revealed that LMIV230‐01 binds to a different face of the antigen compared to a murine mAb elicited via immunization with Pf female gametocytes, mAb 4F12 [200] (Figure 2B,C). Despite this, LMIV230‐01 and mAb 4F12 appear to share similar levels of TRA (IC_80_ = 55 and 48 μg/mL, respectively) [201].

Ivanochko and Fabra‐García et al. [201] structurally characterized four mAbs acquired from natural infection from a Dutch expatriate [202] who lived in Central Africa for approximately 30 years and who had acquired transmission‐blocking immunity. A collection of mAbs from this donor as well as mAbs from an 8‐year‐old Ugandan donor from Tororo [203], were assessed in SMFAs. The anti‐Pfs230D1 mAbs isolated from these donors targeted a variety of surfaces on Pfs230D1, but primarily targeted one of three epitope bins. These include bin I, which correspond to the mAb 4F12 epitope; bin II, which is targeted by LMIV230‐01; or bin III, which consisted of a new epitope class. Surprisingly, bin I binders isolated in this study were significantly more potent than the bin II binders identified from the two donors. Four of the five bin I binders (RUPA‐32, ‐39, ‐55, and ‐97) exhibited IC_80_ values ranging from 0.7 to 3.1 μg/mL. The fifth mAb, RUPA‐103, while exhibiting a lower potency still exhibited potent TRA with an IC_80_ of 10.3 μg/mL. Mirroring what was reported before, these mAbs required complement to achieve potent TRA. In contrast, the five bin II binders tested in SMFAs did not exhibit potent TRA as 100 μg/mL of mAb was insufficient to achieve 80% TRA.

Crystal structures of Pfs230D1 in complex with bin I mAbs, RUPA‐32, ‐55, and ‐97 (Figure 2B,C), revealed that they bound nearly identical epitopes with few unique interactions distinguishing them from one another. While the epitopes are slightly different, RUPA‐32, ‐39, and ‐97 have similar angles of approach to mAb 4F12 (Figure 2C). Ivanochko and Fabra‐García et al. [201] noted that the lower affinity of mAb 4F12 (*K*_D_ = 25 nM) to Pfs230D1 compared to the RUPA‐32, ‐55, and ‐97 (*K*_D_ = 2.5–6.7 nM) might explain the difference in potency (48 μg/mL vs. 0.7–2.8 μg/mL, respectively). Interestingly, Tang and Coelho et al. [187] also characterized mAb 230AL‐18 elicited via Pfs230D1‐EPA immunization (Figure 2C), which bound with a nearly identical angle of approach as bin I mAb, RUPA‐97, and displayed TRA above 80% at 100 μg/mL.

One of the bin II binders, RUPA‐38, was also crystallized in complex with Pfs230D1, revealing that it shared a similar binding interface as LMIV230‐01 [199] (Figure 2B,C). However, the antibodies bound through different angles of approach, which might be a contributing factor to different potencies observed as RUPA‐38 failed to exhibit TRA > 80% in SMFAs even at 100 μg/mL. Tang and Coelho et al. [187] have also structurally characterized mAbs elicited via Pfs230D1‐EPA immunization that bind outside of these two bins—some of these also displaying appreciable TRA (Figure 2B,C).

Crystal structures of murine‐derived mAb 15C5 [204] and human‐derived LMIV230‐02 [199] were also solved in complex with Pfs230D1 that bound a distinct face from the bin I and II sites and named bin III (Figure 2D). Both of these mAbs were elicited through immunization with recombinant Pfs230D1 but lacked any functional TRA. These mAbs shared only a small portion of their epitope with each other but were similar in that they engaged with the C‐terminal portion of Pfs230D1 (Figure 2D). Through AlphaFold 2 modeling, Ivanochko and Fabra‐García et al. [201] revealed that the position of the mAbs overlap with the position of Pfs230 domain 2, suggesting that the epitope was not accessible and may contribute to the lack of TRA observed. The structure was validated when crystal structures of Pfs230 construct containing domains 1 and 2 were solved [187, 188]. In support of this hypothesis, both mAbs 15C5 and LMIV230‐02 do not bind to *P. falciparum* gametes in immunofluorescent assays [199, 201]. Findings in two other studies [187, 188] further support this proposed model. Dietrich et al. [188] found that immunizing an alpaca with a Pfs230D1D2 construct, which does not display the bin III surface, yielded nanobodies that only bound to two competition groups. Crystal structures of one representative nanobody from each of these two groups (nanobody F5—bin II, nanobody F10—bin I) in complex with Pfs230D1 confirmed that bin III nanobodies were not elicited. Furthermore, Tang and Coelho et al. [187] found that mAb 230AS‐26 [187] (Figure 2D) elicited via Pfs230D1‐EPA immunization did not bind live gametes and also displayed poor TRA below 80% in SMFAs at 100 μg/mL. This antibody also binds proximally to the C‐terminus of Pfs230D1. Therefore, it is likely that bin III mAbs target an epitope not exposed on the surface of endogenous Pfs230. The current evidence indicates that angle of approach, high affinity, complement‐fixing subclass, and ability to bind full‐length Pfs230 on live gametes are integral features of Pfs230D1‐targeting mAbs for functional TRA.

### 4.2 Antibodies Against Pfs48/45

**FIGURE 3:**
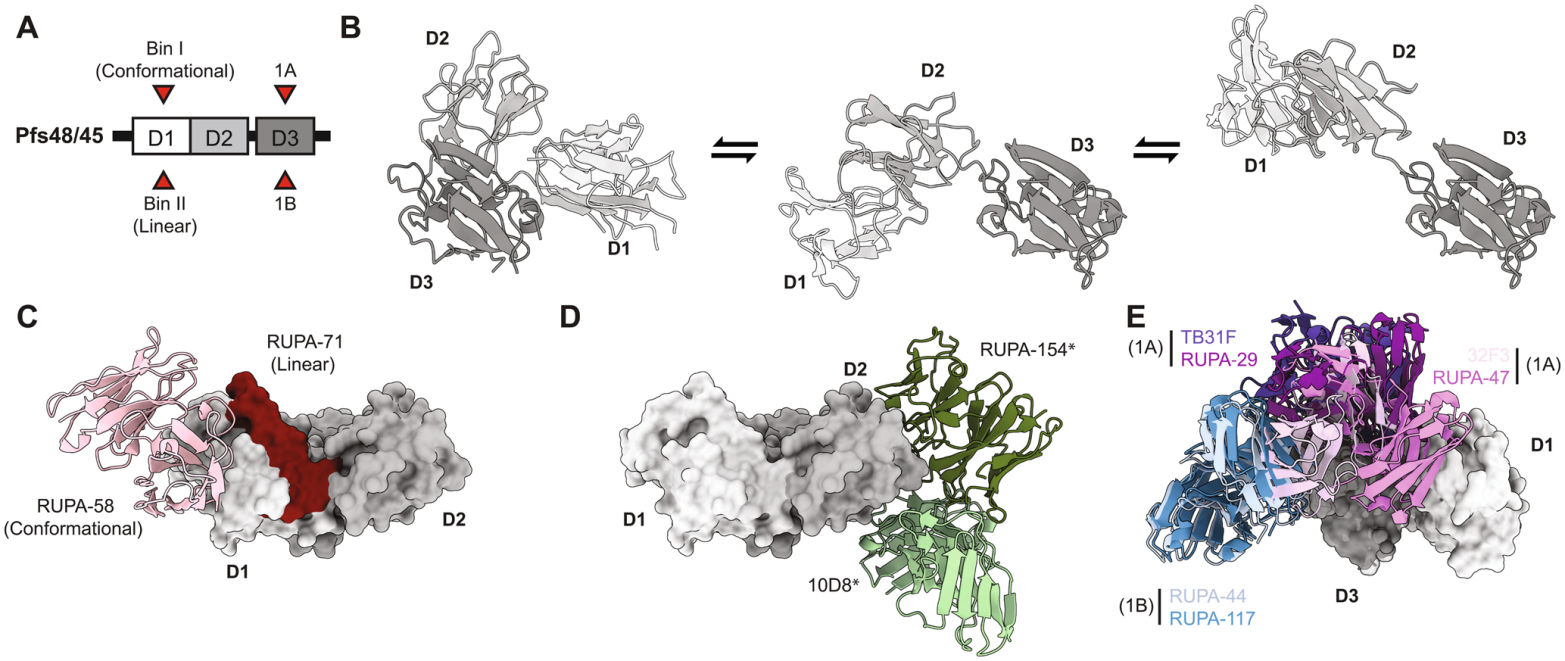
Overview of structurally characterized Pfs48/45‐directed mAbs. (A) Domain architecture of Pfs48/45 is shown with boxes indicating each 6‐Cysteine domain. Red arrows indicate epitopes that primarily induce highly potent mAbs with an IC_80_ < 10 μg/mL. (B) Different conformations adopted by Pfs48/45 which range from compact discs to extended. Presumably, the flexibility observed is a result of D1D2 existing as a tandem domain [186] which is separated from D3 by a flexible linker. (C) Characterized epitopes of Pfs48/45D1 antibodies include bin I (conformational) depicted by RUPA‐58 (pink) and bin II (linear) determined through HDX‐MS [207]. (D) Structurally characterized antibodies that target Pfs48/45D2 include RUPA‐154 (dark green) and mAb 10D8 (light green). Asterisks next to the antibody indicates that they are poorly inhibitory (IC_80_ > 100 μg/mL). (E) Two potent epitopes have been structurally characterized for antibodies that target Pfs48/45D3: 1A: mAb TB31F (purple), mAb RUPA‐29 (violet), mAb 32F3 (light pink), and mAb RUPA‐47 (pink); and 1B: mAb RUPA44 (light blue) and mAb RUPA‐117 (dark blue). 1A‐targeting mAbs, TB31F and RUPA‐29 exhibit IC_80_ ~ 1–2 μg/mL [208, 209], whereas mAbs 32F3 and RUPA‐47 are significantly less potent [208, 210].

The other TBV target shown to elicit antibodies of similar potency to the Pfs230D1 bin I mAbs [201] is Pfs48/45. Unlike Pfs230‐directed mAbs, Pfs48/45 mAbs can act through a neutralization mechanism that can be independent of complement [205]. Pfs48/45 forms a heterodimer complex with Pfs230 [184] and is expressed on the surface of sexual stage parasites through a C‐terminal GPI anchor [206]. Pfs48/45 is composed of three 6‐Cysteine domains (Figure 3A). Unlike the majority of other Pf proteins that harbor these domains as tandem pairs, Pfs48/45 contains an odd number of domains resulting in one of the domains not being structurally coupled to another [186]. Full‐length structures of Pfs48/45 in complex with Fab domains of antibodies have been reported by Ko, Lennartz, and Mekhaiel et al. [210] and Kucharska, Ivanochko, and Hailemariam et al. [207] through crystallography and cryogenic electron microscopy, respectively. A range of conformations were observed—from a compact disc‐like conformation to a largely extended conformation (Figure 3B). In the extended conformation, the tandem A‐type and B‐type 6‐Cysteine pair corresponds to domains 1 and 2 and as such, the position of domain 3 appears to be more variable, adopting different interdomain angles in structures determined by cryogenic electron microscopy (Figure 3B).

Potent antibodies targeting domain 1 of Pfs48/45 (Pfs48/45D1) were isolated from a Dutch expatriate [202]. While not all mAbs isolated against Pfs48/45D1 were potent, several antibodies isolated against this domain displayed TRA > 80% at 10 μg/mL [207, 208]. Through biolayer interferometry (BLI)‐based competition assays, two potent epitope bins were identified [207]—a conformational epitope, Pfs48/45D1 bin I; and a linear epitope [208], Pfs48/45D1 bin II. The Pfs48/45D1 bin I epitope was structurally characterized by cryogenic electron microscopy in which full‐length Pfs48/45 was bound to a potent (IC_80_ =  7.5 μg/mL) [207] bin I targeting mAb, RUPA‐58 [207] (Figure 3C). While the linear epitope [208] Pfs48/45D1 bin II has yet to be structurally characterized, a series of biophysical experiments, mutagenesis, and hydrogen‐deuterium exchange mass spectrometry delineated the interface targeted by the Pfs48/45D1 bin II antibodies, revealing that it binds a region proximal to the bin I epitope (Figure 3C). Interestingly, in the IgG format, an antibody against this bin displayed nanomolar apparent affinity to both Pf and Pv48/45, suggesting that it could provide transmission reducing activity against both *Plasmodium* species. Other mAbs that do not fall into either epitope bins have also been identified [207] with little functional activity. Thus, while the two epitopes described here appear to be Pfs48/45D1 surfaces targeted by potent antibodies, more work is required to gain a mechanistic understanding of what drives Pfs48/45D1‐directed mAb potency and fully delineate all potent epitopes on Pfs48/45D1.

In contrast to Pfs48/45D1, domain 2 of Pfs48/45 (Pfs48/45D2) seems to be associated with lower antibody potency [192, 208, 211, 212]. Two mAbs with distinct competing epitopes (mAbs 10D8 [210] and RUPA‐154 [207]) targeting Pfs48/45D2 have been structurally elucidated; however, these mAbs lack potent TRA (mAb 10D8 with TRA < 80% at 350 μg/mL and mAb RUPA‐154 with an IC_80_ = 103.1 μg/mL) (Figure 3D). Only one Pfs48/45D2‐directed mAb, RUPA‐160 [208], was found to display TRA on par with antibodies targeting domains 1 and 3 of Pfs48/45.

Predating the discovery of highly potent human mAbs [201, 208], the rat‐derived mAb, 85RF45.1 [192]—which targets domain 3 of Pfs48/45 (Pfs48/45D3) and was elicited from Pf gametocyte immunization—was the most potent mAb with TRA. The epitope was structurally characterized by Kundu and Semesi et al. [213] and Lennartz and Brod et al. [214] and was initially referred to as epitope 1 [192] (a nomenclature then changed to epitope 1A [208]). The Fab fragment of the mAb was crystalized in complex with Pfs48/45D3, sometimes referred to as “6C”. While the conformational epitope of mAb 85RF45.1 contains documented polymorphisms, BLI experiments utilizing Pfs48/45D3 constructs harboring these mutations revealed that nanomolar affinity was still maintained independent of amino acid residues at these positions. To advance the mAb into clinical trials to evaluate its safety, tolerability, and transmission‐reducing activity [4], Kundu and Semesi et al. [209] humanized mAb 85RF45.1 to generate mAb TB31F (Figure 3E). A crystal structure of this mAb in complex with Pfs48/45D3 revealed that it bound very similarly to parental mAb 85RF45.1 and maintained nanomolar level affinity binding. Additionally, the TB31F Fab exhibited better biophysical properties in comparison to 85RF45.1 Fab with a melting temperature of (*T*_m_) 74.8°C and an aggregation temperature (*T*_agg_) of 73.0°C, compared to 63.1°C and 63.8°C for 85RF45.1 Fab, respectively. Both mAbs when tested in SMFA yielded IC_80_ values of approximately 1 μg/mL.

From the same donor in which the highly potent Pfs230D1 bin I mAbs [202] were isolated (Dutch expatriate), Fabra‐García, Hailemariam, and de Jong et al. [208] isolated RUPA‐29. This antibody was structurally delineated to bind similarly to mAb TB31F as the two mAbs bind Pfs48/45D3 sharing largely overlapping binding surfaces (Figure 3E). Importantly, both mAbs display similar levels of potency, as RUPA‐29 displayed 80% TRA at 2 μg/mL [202]. In addition to mAb RUPA‐29, three other mAbs (RUPA‐50, RUPA‐54, and RUPA‐100) that share genetic similarity to mAb RUPA‐29, were all on the same level of potency. However, not all mAbs that target this epitope display the same level of TRA (Figure 3E). mAb RUPA‐47 when tested at 10 μg/mL in SMFAs, was not able to achieve TRA above 80% which may be explained by the slightly different binding surface targeted by the mAb (Figure 3E). Additionally, murine‐derived mAb 32F3 elicited through gametocyte immunization [192], which was structurally determined to target the same epitope bin [210], only displayed TRA > 80% in SMFA when tested at 375 μg/mL [211] (Figure 3E). Therefore, while this epitope is the target of highly potent mAbs, it can also elicit antibodies of more variable potencies. Future studies that unravel the molecular basis of these differences in potencies will be important to guide immunogen design efforts to preferentially elicit TB31F‐like mAbs over those with moderate or lower potency.

Fabra‐García, Hailemariam, and de Jong et al. [208] also structurally demonstrated that Pfs48/45D3 is targeted by antibodies to a non‐competing epitope. mAbs RUPA‐44 and RUPA‐117, which are genetically similar antibodies, bind nearly identically to Pfs48/45D3, targeting an interface on the opposite side of epitope 1A (Figure 3E). Epitope 1B is targeted by potent antibodies with TRA > 80% in SMFAs when tested at 10 μg/mL. Like mAb TB31F, common polymorphisms observed in epitope 1B do not perturb the binding affinity of mAbs RUPA‐117 and RUPA‐44 substantially. Epitope 1B‐targeting mAbs are likely significant contributors to the potent Pfs48/45D3‐directed humoral response. Indeed, mice immunized with Pfs48/45D3‐based immunogens that perturb this epitope through glycan‐masking displayed comparatively poorer TRA compared to mice that were immunized with non‐glycan‐masked Pfs48/45D3 immunogens [215].

Pfs48/45D3 immunization studies have demonstrated that inducing potent humoral immune responses against this domain can be challenging [206, 211, 213, 216]. McLeod et al. [215] hypothesized that improving Pfs48/45D3 antibody responses could be accomplished by structure‐guided stabilization of Pfs48/45D3, allowing the optimal presentation of potent epitopes. Drawing on principles applied in the context of the design of respiratory syncytial virus (RSV) [214] and coronoavirus disease 2019 (COVID‐19) [217] vaccines, the researchers identified key mutations that greatly stabilized the conformational of Pfs48/45D3 recognized by potent mAb TB31F. Certain combinations of these mutations yielded immunogens with superior biophysical properties improving the *T*_m_ of Pfs48/45D3 from 46.9°C ± 2.5°C to 69.8°C‐72.3°C. This was accomplished while maintaining the epitope 1A surface, evident by nanomolar‐level affinity binding of TB31F to these stabilized immunogens. A structure of one of these immunogens, 6C.mAgE1 crystallized in complex with Fabs, revealed the structural basis for improved biophysical properties. Steric alleviation, improved van der Waal packing, and introduction of a hydrogen bond collectively contributed to the higher thermostability of these immunogens. Notably, McLeod et al. [215] demonstrated that Pfs48/45D3 stabilization could significantly bolster the functional immune response. Immunizing CD‐1 mice with these immunogens presented on the CoPoP multimerization platform [218] yielded potent TRA with purified total IgGs exhibiting IC_80_s in SMFAs ranging from 33.7 to 57.7 μg/mL. In contrast, 1500 μg/mL of IgG purified from the serum of mice immunized with WT Pfs48/45D3 displayed on the CoPoP multimerization platform [218] did not yield TRA > 80% (62.7%). Stabilization of Pfs48/45D3 appears to be a viable approach to induce high quality responses against this malarial antigen, as similar findings were described by Dickey et al. [219] where Pfs48/45D3 stabilization utilizing a different set of mutations yielded an improved immunogen compared to the WT antigen.

## 5 Future Perspectives

As *Plasmodium* parasites have coevolved alongside humans for millions of years [220], they have developed a plethora of immune evasion strategies [221] that must be overcome for the development of effective biomedical interventions. Some have proposed that the repeat region of CSP may function as a decoy by promoting suboptimal immune responses [96]. Through its immunogenic repetitive motifs, the central region of CSP has been hypothesized to promote inefficient germinal center reactions [98] and drive primarily T cell‐independent responses [164]. Indeed, multivalent binding of multiple B cell receptors expressed on a single B cell may, through avidity effects, permit the activation of B cells even when the affinity to the antigen is low. Evidence for this lies in the enrichment of somatically hypermutated residues at homotypic interaction interfaces of many CSP‐directed mAbs [116, 118, 119, 120, 121, 122, 135]. Kucharska et al. [222, 223] have demonstrated that this phenomenon is not exclusive to PfCSP and extends to CSP molecules expressed by *Plasmodium vivax* (Pv) and *Plasmodium berghei* (Pb). Murine antibodies elicited against PvCSP and PbCSP, despite being composed of different amino acid repeats than PfCSP, also select for mutations that strengthen the homotypic interaction interface, suggesting a generalizable property of the mammalian immune response against repeat antigens [135, 222, 223]. Striking the right balance in the number and composition of repeating elements may be critical for the elicitationof sustained protection by vaccines.

Interestingly, sequences found in the repeats of PfCSP have also been found in antigens expressed in other life cycle stages [224]. This phenomenon is not unique to CSP. Pf is notorious for expressing a wide range of repetitive elements oftentimes shared across different proteins expressed in different life cycle stages [225]. Multiple antibodies have been reported on that are cross‐reactive to different repeat elements expressed in the *Plasmodium* proteome. Amen, Yoo, and Fabra‐García et al. [226] characterized one of these mAbs, B1E11K, that is cross‐reactive to repeat elements rich in glutamate, present in asexual (RESA) and sexual stage antigens (Pfs230 and Pf11.1). It is unclear as to how cross‐reactivity of repeat antigens across life stages shapes the immune response. More work in this regard will help better understand immune responses in natural infection and also how vaccine responses might be boosted upon parasite exposure.

In regard to transmission‐blocking vaccines, despite their conceptualization in the 1970s [169, 170], the precise molecular functions of various lead TBV protein antigens are not well defined. Obtaining a more in‐depth understanding of the functional role of these proteins could directly translate to the design of more potent vaccines [214]. Thus, research into deciphering the function of these proteins should be prioritized. Most notably, it will be crucial to structurally elucidate the full‐length structure of the Pfs230‐48/45 heterodimer complex as it could help decipher mechanistic insights into what drives antibody potency. Indeed, it currently remains unclear why some antibodies are more potent than others despite binding similar surfaces (Figures 2B,C and 3E).

Targeting bottlenecks in transmission is a viable strategy toward malaria elimination goals. However, not discussed in this review are the strides being made toward the development of vaccines outside the transmission bottlenecks, such as effective blood stage vaccines. Indeed, there is a strong incentive to develop a diverse panel of malaria vaccines that can contribute to a variety of disease reduction and elimination goals [6, 7]. The development of an effective blood stage vaccine has been challenging as invasion proteins partake in redundant function or display a high degree of polymorphism [227]. PfRH5, an essential protein for erythrocyte invasion [38], contrasts other blood stage vaccine targets in this regard as it is well conserved. RH5.1 [228], a variant of PfRH5, is adjuvanted with Matrix‐M in clinical evaluation [229, 230] and has shown to be safe, immunogenic, and efficacious against clinical malaria in children [230]. The continued development and clinical evaluation around PfRH5 (and the PfRCR complex more broadly) is an exciting area of current and future research for the malaria field, also as it pertains to the continued isolation and characterization of potent antibodies to guide next‐generation efforts [39, 231, 232, 233].

With two effective pre‐erythrocytic vaccines approved for use, as well as promising clinical trials from RH5.1/Matrix‐M [229, 230] and Pfs230D1‐EPA/Alhydrogel [234], the field is well positioned to begin conceptualization, development, and evaluation of multistage malaria vaccines. Combining antigens presented at multiple stages of the parasite's life cycle would be advantageous in that it could be more impactful and cost‐effective in certain contexts [7, 82]. Indeed, evidence suggests that synergistic effects [83] could be obtained combining antigens from different stages. Multi‐stage vaccine immunogens designed to integrate multiple antigens—such as those that have been the focus of this review (CSP, Pfs48/45, and Pfs230)—into a single molecule have begun undergoing clinical evaluation [235, 236, 237] with some showing promising early results. Advancements in RNA delivery technologies are also enabling the design of multicomponent RNA vaccine formulations [238, 239, 240, 241] that incorporate several antigens. It will be of great interest to evaluate in the years to come how these technologies can be best deployed against malaria to target sites of vulnerability across parasite life cycle stage antigens.

Of particular importance should be focusing future research on targets that might have the potential to expand beyond Pf alone, and move toward cross‐species biomedical interventions. While this is a relatively unexplored area of research, some targets have shown early potential as cross‐species targets. Feng et al. [242] reported on a panel of murine mAbs raised against a PbHAP2 domain 3 fragment that displayed cross‐reactivity to a panel of Plasmodium HAP2 domain 3 variants, showing potential as a pan‐Plasmodium antigen. Indeed multiple groups have reported on the strong potential of utilizing HAP2 as a TBV in the context of various Plasmodium species with the regions between species showcasing strong sequence homology [243, 244, 245, 246]. However, structural characterization of antibodies against HAP2 have yet to be conducted and should be an area of future work. Aside from HAP2, Tang et al. [247] recently reported on two antibodies that were cross‐reactive to both Pv and Pf variants of the cell‐traversal protein for ookinetes and sporozoites (CelTOS) protein, a proteinwhich is expressedimplicated in both pre‐erythrocytic and sexual stages. The fact that most of these targets have been reported on in recent years further exemplifies the importance of continuing antigen discovery efforts as many unidentified or uncharacterized epitopesantigens could hold promise as next‐generation vaccine candidates.

In parallel, the most potent and broad mAbs identified in discovery efforts can be directly used in passive immunization, which has been an active area of development in recent years (mAbs CIS43LS, L9LS, MAM01, and TB31F) [89, 90, 91, 93]. For these mAbs to add to the armamentarium of biomedical interventions against malaria and become accessible, they will need to achieve high potencies to become economically viable. Methods to lower the cost of manufacturing, and/or improve the potency of current mAbs are thus an interesting area of research to determine what can propel potency, resilience against escape and the possibility of combining multiple specificities in a single product [92, 248, 249, 250, 251]. While combating malaria has been a daunting challenge, the wealth of knowledge that has been acquired in the past decade has enabled the development of exciting tools to combat malaria. With sustained support, large strides will continue to be made in the years to come, aspirationally enabling malaria elimination and eradication goals.

## Conflicts of Interest

The Hospital for Sick Children has filed intellectual property related to molecules described in this review, with JPJ as a co‐inventor. All other authors declare no conflicts of interest.

## Data Availability

The structural data analyzed in this study are openly available in the RCSB Protein Data Bank (https://doi.org/10.2210/pdbXXXX/pdb), where XXXX corresponds to PDB ID identifiers.
